# Supplementary material for: Modelling filovirus maintenance in nature by experimental transmission of Marburg virus between Egyptian rousette bats
Source: Nat Commun. 2017 Feb 13;8:14446. doi: 10.1038/ncomms14446 (PMC5316840; doi:10.1038/ncomms14446)
Supplement: Supplementary Information — Supplementary Tables [file ncomms14446-s1.pdf]

Supplementary Table 1 | No difference in MARV RNA loads, duration of MARV RNA detection and peak MARV IgG antibody levels according to sex.

	Males					Females						
		Descriptive statistics		D'Agostino & Pearson			Descriptive statistics		D'Agostino & Pearson		Mann-Whitney U	
Parameter	N	Mean	SD	K2	p	N	Mean	SD	K2	p	U	p
Inoculated donor bats, MARV RNA loads ($log_{10}TCID_{50}$eq mL^{-1}), blood	35	2.0	2.3	55.07	<0.0001	34	1.9	2.2	63.9	0.0001	575.0	0.8161
Inoculated donor bats, MARV RNA loads ($log_{10}TCID_{50}$eq mL^{-1}), oral swabs	24	4.1	4.8	59.27	<0.0001	27	4.1	4.6	64.25	<0.0001	284.0	0.4593
Inoculated donor bats, MARV RNA loads ($log_{10}TCID_{50}$eq mL^{-1}), rectal swabs	2	NNS	NNS	NNS	NNS	4	NNS	NNS	NNS	NNS	NA	NA
Inoculated donor bats, duration of MARV RNA detection (days), blood	7	5.0	2.8	NNS	NNS	5	7.0	2.2	NNS	NNS	NA	NA
Inoculated donor bats, duration of MARV RNA detection (days), oral swabs	7	3.4	3.3	NNS	NNS	5	5.4	3.4	NNS	NNS	NA	NA
Inoculated donor bats, duration of MARV RNA detection (days), rectal swabs	7	0.3	0.8	NNS	NNS	5	0.8	0.8	NNS	NNS	NA	NA
Inoculated donor bats, peak MARV IgG antibody levels (sum OD)	7	2.9	1.0	NNS	NNS	5	3.7	1.0	NNS	NNS	NA	NA
Naïve contact bats, peak MARV IgG antibody levels (sum OD)	3	NNS	NNS	NNS	NNS	6	1.9	0.8	NNS	NNS	NA	NA

NNS: Number not sufficient, NA: Not appropriate.

Supplementary Table 2 | Urine collection attempts from inoculated donor bats.

	DPI																													
**Bat**	1	2	3	4	5	6	7	8	9	10	11	12	13	14	15	16	17	18	19	20	21	22	23	24	25	28	35	42	49	56
214605 (1 ID)																														
220235 (1 ID)																														
220599 (1 ID)																														
550417 (1 ID)																														
642999 (3 ID)																														
656429 (3 ID)																														
685891 (3 ID)																														
685787 (3 ID)																														
685734 (5 ID)																														
686146 (5 ID)																														
691198 (5 ID)																														
720561 (5 ID)																														

Times shaded in gray indicate successful urine collection attempts and times shaded in black indicate MARV RNA positive specimens. The housing unit (1-6) and group (inoculated donor-ID) during the early study phase is indicated following the identification number of each bat.

Supplementary Table 3 | Primers and probes.

Name	Sequence (5' to 3')
**Marburg virus viral protein 40 gene**	
Forward primer	GGA CCA CTG CTG GCC ATA TC
Reverse primer	GAG AAC ATI TCG GCA GGA AG
Probe 1	*56-FAM*-ATC CTA AAC-*ZEN*-AGG CTT GTC TTC TCT GGG ACT T-*3IABkFQ*
Probe 2	*56-FAM*-ATC CTG AAT-*ZEN*-AAG CTC GTC TTC TCT GGG ACT T-*3IABkFQ*
**Rift Valley fever virus large segment**	
Forward primer	TGA AAA TTC CTG AGA CAC ATG G
Reverse primer	ACT TCC TTG CAT CAT CTG ATG
Probe	*FAM*-CAC AAG TCC ACA CAG GCC CCT TAC ATT G-*BHQ1*
